# Evidence of biological activity of *Mentha* species extracts on apoptotic and autophagic targets on murine RAW264.7 and human U937 monocytic cells

**DOI:** 10.1080/13880209.2016.1235208

**Published:** 2016-12-07

**Authors:** Fatiha Brahmi, Samia Hadj-Ahmed, Amira Zarrouk, Maryem Bezine, Thomas Nury, Khodir Madani, Mohamed Chibane, Anne Vejux, Pierre Andreoletti, Lila Boulekbache-Makhlouf, Gérard Lizard

**Affiliations:** a Team 'Biochemistry of the Peroxisome, Inflammation and Lipid Metabolism’ EA 7270/INSERM, Faculté des Sciences Gabriel, University Bourgogne Franche Comté, Dijon, France;; b Laboratory of 3BS, Faculty of Life and Nature Sciences, Universty of Bejaia, Bejaia, Algeria;; c Faculty of Medicine, LR12ES05, Lab-NAFS ‘Nutrition – Functional Food & Vascular Health’, University Monastir, Monastir, Tunisia;; d Laboratory Biochemistry, Faculty of Medicine, University of Sousse, Sousse, Tunisia;; e Laboratory of ‘Venoms and Therapeutic Biomolecules’, University of Tunis El Manar – Pasteur Institut, Tunis, Tunisia;; f Faculty of Life, Nature and Earth Sciences, University of Akli Mohand Oulhadj, Bouira, Algeria

**Keywords:** *Mentha spicata*, *mentha pulegium*, *mentha rotundifolia*, mitochondria

## Abstract

**Context:** Mints (Lamiaceae) are used as traditional remedies for the treatment of several diseases. Their extracts are recognized as anti-inflammatory compounds.

**Objective**: This study characterized the cytotoxic effects of *Mentha spicata* L. (MS), *Mentha pulegium* L. (MP) and *Mentha rotundifolia* (L). Huds (MR) on macrophage cells (RAW264.7; U937) and determined their impact on apoptosis and autophagy, which can play a role in controlling inflammation.

**Materials and methods:** The extracts were prepared in culture medium and tested from 25 to 400 μg/mL after 24–48 h of treatment. To show the effect of the aqueous ethanol (50%) extracts on apoptosis and authophagy, the presence of cleaved caspase-3, and the conversion of LC3-I to LC3-II was evaluated by Western blotting.

**Results:** Compared with the MTT assay, crystal violet showed a pronounced decrease in the number of cells with all extracts at 48 h. Calculated IC_50_ values were 257.31, 207.82 and 368.02 μg/mL for MS, MP and MR, respectively. A significant increase in PI positive cells was observed with all extracts at 200-400 μg/mL. Mitochondrial dysfunctions and nuclear morphological changes were detected with MS and MR extracts at 400 μg/mL. At this concentration, no cleaved caspase-3 was found whereas stabilized caspase-3 in its dimeric form was identified. MS and MR extracts also favour LC3-I to LC3-II conversion which is a criterion of autophagy.

**Conclusions:** The cytotoxic profiles depend on the extracts considered; MS extract showed the strong activity. However, all the mint extracts studied interact with the apoptotic and autophagic pathways at elevated concentrations.

## Introduction

The genus *Mentha* comprises 61 species according to the latest taxonomic treatment and is among the major genera belonging to the Lamiaceae family (Brahmi et al. 2015). Members of the mint family are found throughout the world, but the main centre of distribution is the Mediterranean area, where these plants form a dominant part of the vegetation (Doymaz 2006).

According to the Flora of Algeria (Quezel & Santa 1962), *Mentha* is represented by five major species; *M. spicata* L. (MS), *M. pulegium* L. (MP) and *M. rotundifolia* (L.) Huds (MR) are the most prevalent. These species have been used in different traditional medical systems as herbal remedies but are also commonly used in the Mediterranean diet as herbal teas and spices (Lopez et al. 2010). MS has many medicinal uses: biliary disorders, menstrual cramps, stomach pain, constipation, gingivitis and toothache are treated with the decoction of spearmint leaves; leaves are also used as a poultice to relieve rheumatism and combat fever (Brahmi et al. 2014). The aerial part of MP has traditionally been used to treat flatulent dyspepsia, intestinal colic, amenorrhea, gout, and colds. MR is widely used in Algerian traditional medicine to treat furunculosis and abscesses, used as a friction rub it is said to reduce fever (Brahmi et al. 2016). 

The historical use of *Mentha* species is no different from its use in modern herbal medicine. The antimicrobial, antioxidant, anti-inflammatory, neuroprotective, cardiovascular and antitumor properties of mint extracts have also been shown (Bello et al. 2001; Mata et al. 2007; Hussain et al. 2010; Pearson et al. 2010).

Recently, a variety of plant extracts have been investigated for their ability to influence the apoptotic process. Apoptosis (also named programed cell death) can be defined as a mechanism characterized by a series of different morphological and biochemical changes, including an increased reactive oxygen species (ROS) level, the activation of caspases, cell shrinkage, chromatin condensation and nucleosomal degradation (Forbes-Hernandez et al. 2014). Apoptosis is induced by many biological, chemical, and physical agents, and it is involved in embryonic development (Lizard et al. 1995).

Overproduction of ROS deregulates apoptosis and leads to cancer, cardiovascular diseases and many neurodegenerative disorders. Hence, a balance between ROS formation and antioxidant activity are essential for the normal functions of the body.

The use of novel dietary compounds that directly affect mitochondrial functionality has the potential to emerge as a key platform technology for the next generation of functional foods, nutraceuticals and drugs (Forbes-Hernandez et al. 2014). *Mentha* species that proved to be a potent source of antioxidants in our previous studies (Brahmi et al. 2014, 2015) were chosen to evaluate their action on the apoptotic pathway especially at the mitochondrial level and on caspase-3 activation on murine RAW264.7 and human U937 monocytic cells. As apoptosis can be connected to autophagy, the impact of *Mentha* extracts on LC3-I to LC3-II conversion, which is a criterion of autophagy, was also determined.

To our knowledge, no documented study has investigated the ability of *Mentha* species extracts to trigger apoptosis on macrophages, which play major roles in the inflammation process associated with major inflammatory diseases.

As *Mentha* extracts are recognized in traditional medicine as antipyretic and anti-inflammatory compounds, and as macrophages play key roles in these processes in several acute and chronic inflammatory diseases, the main objectives of the present study consisted in characterizing the cytotoxic effects of three *Mentha* species [*M. spicata* (MS), *M. pulegium* (MP) and *M. rotundifolia* (MR)] on macrophage cells (RAW264.7; U937), especially to determine their impact on the main targets of apoptosis and autophagy, which play key roles in the control of inflammatory status (Headland & Norling 2015; Viola & Soehnlein 2015).

## Materials and methods

### Herbal material

The fresh leaves of cultivated *M. spicata*, and wild *M. pulegium* and *M. rotundifolia* were collected from the Smaoun region (36°37'0”N, 4°48'0”E) of Bejaia, Algeria in June 2013. Samples were identified by B. Seddik and M. Ourari, botanists at the University of Bejaia, Algeria, by comparing them with voucher specimens previously harvested and deposited in the Herbarium of the National Botanical Garden of Meise (Belgium), references BR 0000006946227 for *Mentha spicata*, BR 0000006946043 for *Mentha pulegium*, and BR 000000 6946197 for *Mentha rotundifolia*.

### Preparation of plant extracts

The leaves were air dried at room temperature to get constant weights. The dried material was then comminuted into coarse powder using the Waring commercial laboratory blender. A known quantity of the powder (20 g) was extracted by stirring with 1000 mL of aqueous ethanol (50%), at room temperature and at 130 rpm for 24 h. The crude extract was filtered on cellulose, concentrated with a rotary vacuum evaporator (40 °C), lyophilized and maintained in the dark at +4 °C until tested. The extracts were diluted in culture medium to prepare the required final concentrations (25, 50, 100, 200, and 400 μg/mL).

### Cells and cell treatments

The murine RAW264.7 cells and the human monocytic U937 cells used were obtained from the European Collection of Animal Cell Cultures (ECACC, Salisbury, UK). RAW264.7 cells were seeded at 240,000 cells per well in 24-well microplates containing 0.5 mL of culture medium (480,000 cells/mL), constituted by Dulbecco’s Modified Eagle Medium (DMEM) (Lonza) supplemented with 5% (v/v) heat-inactivated foetal bovine serum (FBS) (Pan Biotech) and 1% antibiotics (100 U/mL penicillin, 100 μg/mL streptomycin) (Pan Biotech).

Human promonocytic U937 cells (500,000 cells/mL) were grown in RPMI 1640 with GlutaMAX I (Invitrogen, Eragny, France) and antibiotics (100 U/mL penicillin, 100 μg/mL streptomycin; Invitrogen, Cergy-Pontoise, France) supplemented with 10% (v/v) heat-inactivated foetal calf serum (Invitrogen).

RAW264.7 and U937 cells were incubated at 37 °C in a humidified atmosphere containing 5% CO_2_, and passaged twice a week.

### Analysis of cell growth by phase-contrast microscopy

Cell growth was observed after 24 and 48 h of treatment with different *Mentha* extracts used at different concentrations (25, 50, 100, 200, and/or 400 μg/mL) under an inverted phase-contrast microscope (Axiovert 40CFL, Zeiss). Digitized images were obtained with a camera (Axiocam ICm1, Zeiss).

### MTT test

The colorimetric assay utilizing MTT dye (3-[4,5-dimethylthiazol-2-yl]-2,5-diphenyltetrazolium bromide) (Sigma Chemical Co., St. Louis, MO) was used to measure cell viability after treatment with mint extracts at different concentrations (25, 50, 100, 200, and/or 400 μg/mL). The number of viable cells was determined by measuring the reduction of MTT dye (by mitochondrial dehydrogenase) in live cells to blue formazan crystals. RAW264.7 cells were seeded in 24-well plates. After 24 or 48 h seeding, the cells were treated with various concentrations of mint extracts. Subsequently, 500 μL of MTT solution (0.1 mg/mL) were added to each well and the cells were incubated for 3 h at 37 °C. The supernatant was discarded and 500 μL of dimethylsulfoxide were added to each well to dissolve the formazan crystals. The optical density of the formazan solution was measured at 570 nm.

### Crystal violet staining

Quantification of adherent cells was estimated by staining with crystal violet staining (Sigma Aldrich, L'Isle d'Abeau Chesnes, France). Cells were seeded in triplicates in 24 well plates and cultured without or with *Mentha* extract (25, 50, 100, 200 or 400 μg/mL) for 24 and 48 h. At the end of treatment, cells were washed with PBS, stained with crystal violet (5 min), and rinsed with water. Absorbance was read at 570 nm after extraction of the dye with 0.1 mol/L sodium citrate in 50% ethanol.

### Measurement of transmembrane mitochondrial potential with DiOC_6_(3) 

Mitochondrial transmembrane potential (ΔΨm) was measured with 3,3-O-dihexyloxacarbocyanine iodide [DiOC_6_(3)] (Life Technologies). Adherent and non-adherent cells were pooled, and stained with DiOC_6_(3) (40 nM). Loss of ΔΨm is indicated by a decrease in green fluorescence collected through a 520/10 nm band pass filter on a Galaxy flow cytometer (Partec). For each sample, 10,000 cells were acquired. Data were analyzed with Flomax (Partec) or FlowJo (Tree 153 Star Inc.) software.

### Measurement of cytoplasmic membrane integrity with propidium iodide

Adherent and non-adherent cells were pooled and stained with propidium iodide (PI; Sigma-Aldrich); 1 μg/mL, 5 min). Cells with altered membrane integrity (cells with damaged cytoplasmic membranes and/or dead cells) were stained with PI (Nury et al. 2014). The cells were analyzed with a Galaxyflow cytometer (Partec). Red fluorescence of PI was detected through a 630 nm long pass filter. For each sample, 10,000 cells were acquired. Data were analyzed with the FlowMax (Partec) or FlowJo (Tree Star Inc.) softwares.

### Evaluation of nuclear morphology with Hoechst 33342

The nuclear morphology of cells cultured with or without *Mentha* extracts was characterized by fluorescence microscopy after staining with Hoechst 33342. It was used to determine the nuclear morphology by fluorescence microscopy under ultraviolet light. In these conditions, dead cells are characterized by condensed and/or fragmented nuclei (apoptotic cells) as well as by swollen nuclei (oncotic/necrotic cells), whereas living cells have round and regular nuclei. Hoechst 33342 (1 mg/mL in distilled water) was added at 1 μg/mL final concentration on cell deposits of approximately 40,000 cells applied to glass slides by cytocentrifugation (5 min, 15,000 rpm) with a cytospin 2 (Shandon). The morphological aspect of the nuclei was observed with an Axioskop fluorescent Zeiss microscope; 300 cells per sample were examined.

### Protein analysis by polyacrylamide gel electrophoresis and Western blotting

Cells were lysed in a Ripa buffer (10 mM Tris–HCl, pH 7.2, 150 mM NaCl, 0.5% Nonidet NP40, 0.5% Na deoxycholate, 0.1% SDS, 2 mM EDTA and 50 mM NaF) in the presence of 1/25 complete protease inhibitor cocktail tablets (Roche Diagnostics Corporation, Mannheim, German) for 30 min on ice. Cell lysates were cleared by 15 min of centrifugation at 20,000 *g*. The protein concentration was measured in the supernatant using bicinchoninic acid solution (Sigma Aldrich). Proteins (50–80 μg) were diluted in loading buffer (125 mM Tris-HCl, pH 6.8, 10% β-mercaptoethanol, 4.6% SDS, 20% glycerol, and 0.003% bromophenol blue), separated on a polyacrylamide SDS-containing gel, and transferred onto a nitrocellulose membrane (Thermo-Scientific, Courtaboeuf, France). After blocking nonspecific binding sites for 1 h with 5% nonfat milk in TBST (10 mM Tris-HCl, 150 mM NaCl, 0.1% Tween 20, pH 8), the membrane was incubated overnight with the primary antibody diluted in TBST with 1–5% milk and LC3-I/LC3-II (ref L8918, Sigma-Aldrich), and used at a final concentration of 1/1000. Antibody directed against β-actin (Sigma Aldrich, L'Isle d'Abeau Chesnes, France) was used at a final concentration of 1/10,000. The membrane was then washed with TBST and incubated (1 h, room temperature) with horseradish peroxidase-conjugated goat anti-mouse (Santa-Cruz Biotechnology/CliniSciences, Nanterre, France) or anti-rabbit antibody (Santa-Cruz Biotechnology or Cell Signalling) diluted at 1/5000. The membrane was washed with TBST and revealed using an enhanced chemiluminescence detection kit (Supersignal West Femto Maximum Sensitivity Substrate, Thermo-Scientific) and Chemidoc XRS + (Bio-Rad, Marnes la Coquette, France). The level of cleaved caspase-3 determined versus actin, and the ratio [LC3-II/LC3-I] were calculated with Image Lab software (Bio-Rad, Marnes la Coquette, France). 

### Statistical analysis

Statistical analysis was performed using SigmaStat 2.03 software (Systat Software Inc., Chicago, IL) with the Mann–Whitney test. Data were expressed as mean ± SD; data were considered statistically different at a *p*-value of 0.05 or less.

## Results

### Evaluation of the effects of *Mentha* extracts on RAW264.7 cells by phase contrast microscopy, MTT and crystal violet tests

**Figure 1.**
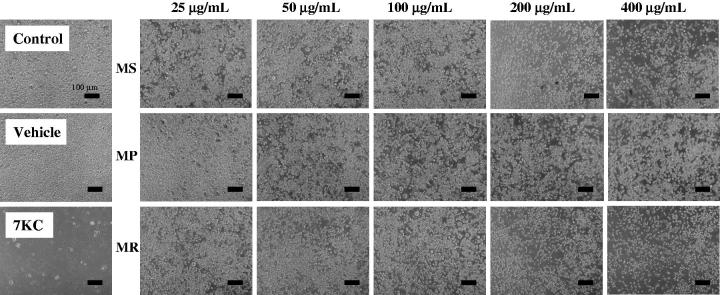
Evaluation of the effect of *Mentha* extracts on cells growth by phase contrast microscopy. The extracts from three Algerian *Mentha* species, *M. spicata* L. (MS)*, M. pulegium* L. (MP), and *M. rotundifolia* (L.) Huds (MR) used at final concentrations of 25, 50, 100, 200, and 400 μg/mL were evaluated on murine RAW264.7 macrophage cells by phase-contrast microscopy after 48 h of treatment. Data shown are representative of at least three independent experiments. 7-Ketocholesterol (7KC; 20 μg/mL) was used as the positive control; vehicle control for 7KC corresponds to ethanol (0.1%).

**Figure 2.**
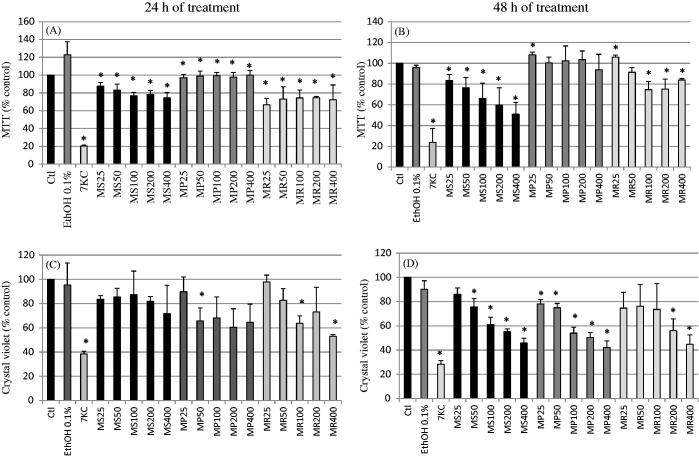
Evaluation of the effect of *Mentha* extracts on cell growth and/or mitochondrial activity with the MTT and crystal violet tests. The extracts from three Algerian *Mentha* species, *M. spicata* L. (MS)*, M. pulegium* L. (MP), and *M. rotundifolia* (L.) Huds (MR) used at final concentrations of 25, 50, 100, 200, and 400 μg/mL were evaluated on murine RAW264.7macrophage cells with the MTT test (A, B) and with the crystal violet test (C, D) after 24 and 48 h of treatment. 7-Ketocholesterol (7KC; 20 μg/mL) was used as the positive control; vehicle control for 7KC corresponds to ethanol (0.1%). Data are mean ± SD from two independent experiments conducted in triplicate. Significance of the difference between untreated- and *Mentha* extract-treated cells (Mann Whitney test; *p < 0.05 or less). No difference was observed between absolute control and vehicle (ethanol: 0.1%).

The treatment of RAW264.7 murine macrophages with *Mentha* extracts (25, 50, 100, 200 and 400 μg/mL) for 24 and 48 h, evaluated by phase-contrast microscopy showed a dose-dependent effect on cell adhesion and cell growth for MS and MR extracts; at 200 and 400 μg/mL, reduced numbers of adherent cells were revealed (Figure 1). With the MTT test, at 24 h of culture, no dose-dependent effects were observed whatever the extract used whereas significant decreases (*p* < 0.05) were observed comparatively to EtOH 0.1% (Figure 2a). At 48 h, whereas no dose-dependent effects were observed with the MP extract, significant dose-dependent effects were observed especially for the MS extract (*p* < 0.05) (Figure 2b). With the crystal violet test, at 24 h, slight not-significant effects were noted with the MS extract (Figure 2c). More pronounced dose effects were found with MP and MR extracts (Figure 2c). At 48 h, a pronounced decrease in the number of adherent cells was detected for all extracts (Figure 2d).

Based on the different data obtained with the MTT and crystal violet tests (Figure 2), only those obtained with crystal violet after 48 h of treatment (Figure 2d) allowed us to simultaneously calculate IC_50_ and IC_10_ values with all mint extracts. Calculated IC_50_ values were: 257.31, 207.82 and 368.02 μg/mL for MS, MP and MR, respectively. Besides, the determined IC_10_ values were: 29.92, 11.69 and 11.49 μg/mL for MS, MP and MR, respectively. Therefore, further experiments were conducted at 200 and 400 μg/mL, which were in the range of IC_50_ values for the different mint extracts considered.

With 7KC, which is a well-known cytotoxic compound, used as positive control, in agreement with the observations made using phase-contrast microscopy, significant effects (*p* < 0.05) were found with the MTT and the crystal violet tests (Figures 1 and 2).

### Evaluation of the effects of *Mentha* extracts on transmembrane mitochondrial potential (ΔΨm) and cytoplasmic membrane integrity of U937 cells

**Figure 3.**
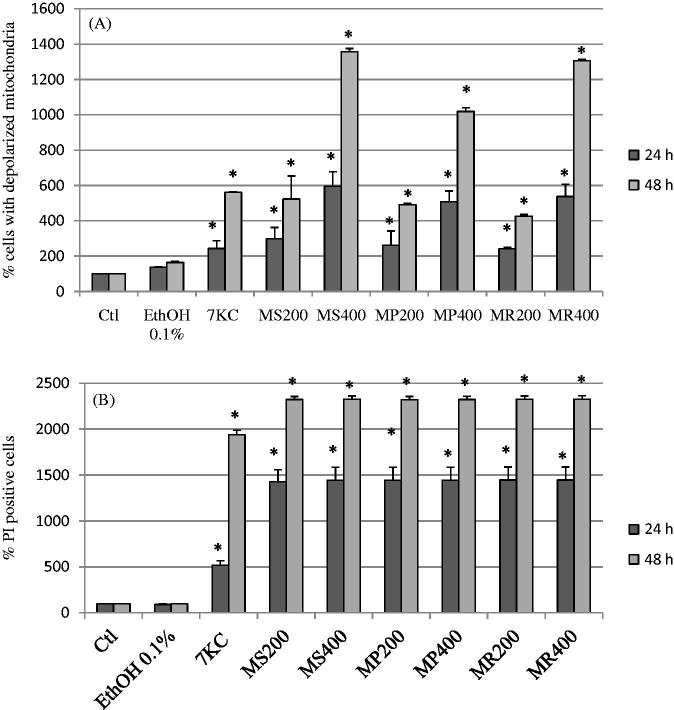
Effects of *Mentha* extracts on mitochondrial transmembrane potential and cytoplasmic membrane integrity. The extracts from three Algerian *Mentha* species, *M. spicata* L. (MS)*, M. pulegium* L. (MP), and *M. rotundifolia* (L.) Huds(MR) used at final concentrations of 25, 50, 100, 200, and 400 μg/mL were evaluated on human U937 monocytic cells after 24 and 48 h of treatment. 7-Ketocholesterol (7KC; 20 μg/mL) was used as the positive control; vehicle control for 7KC corresponds to ethanol (0.1%). Data shown are mean ± SD from two independent experiments conducted in triplicate. Significance of the difference between untreated- and *Mentha* extract-treated cells (Mann Whitney test; *p < 0.05 or less). No difference was observed between absolute control and vehicle (ethanol: 0.1%).

The status of transmembrane mitochondrial potential (ΔΨm) and cytoplasmic membrane integrity provides information on cell viability. Therefore, the effects of mint extracts (200 and 400 μg/mL), which showed the most pronounced effects with the MTT and the crystal violet tests, were determined after 24 and 48 h of treatment on U937 human promonocytic cells. Their effects on ΔΨm and cytoplasmic membrane integrity were measured by flow cytometry after staining with DiOC_6_(3) and PI, respectively. As shown in Figure 3(a), with all extracts, there was a significant dose- and time-dependent increase (*p* < 0.05) in the number of cells with depolarized mitochondria revealed by staining with DiOC_6_(3). After staining with PI, whatever the extract considered, a significant increase (*p* < 0.05) in PI positive cells, corresponding to dead cells or to cells with altered cytoplasmic membranes, was observed at 24 and 48 h of treatment (Figure 3b). It is noteworthy that no fluorescent compounds giving green and red fluorescence, when excited at 488 nm, were detected in all mint extracts (Our data not shown). Therefore, the green and red fluorescence detected by flow cytometry can be considered specific to DiOC_6_(3) and PI, respectively.

With 7KC, used as reference cytotoxic compound, an increase in cells with depolarized mitochondria and PI-positive cells was observed (Figure 3).

### Evaluation of the effects of *Mentha* extracts on the nuclear morphology of U937 cells

**Figure 4.**
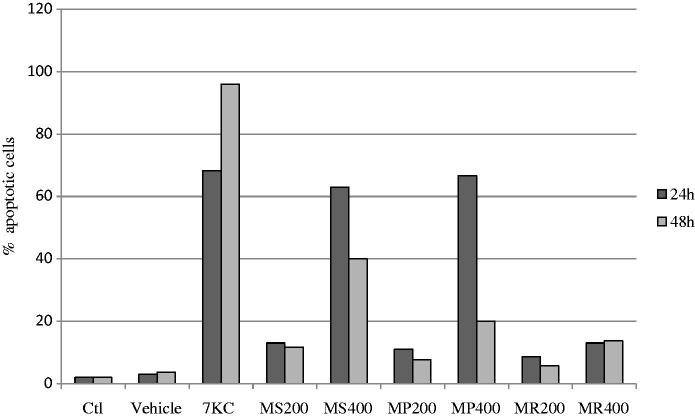
Evaluation of the effects of *Mentha* extracts on apoptosis. The percentage of apoptotic cells (cells with condensed and/or fragmented nuclei) was determined by fluorescence microscopy after staining with Hoechst 33342 on human monocytic U937 cells cultured for 24 and 48 h in the presence *M. spicata* (MS), *M. pulegium* (MP) and *M. rotundifolia* (MR) used at 200 and 400 μg/mL. 7-Ketocholesterol (7KC; 20 μg/mL) was used as the positive control; vehicle control for 7KC corresponds to ethanol (0.1%). Data shown are the mean of two independent experiments.

Nuclei staining with Hoechst 33342 (1 μg/mL) was used to distinguish between viable cells with round and regular nuclei, apoptotic cells with condensed and/or fragmented nuclei, oncotic cells with swollen nuclei, and necrotic cells with irregular and diffuse nuclei of various sizes and shapes (Lizard et al. 1995). When U937 cells were treated with *Mentha* extracts (200 and 400 μg/mL, 24 and 48 h), an increase in the number of apoptotic cells was only observed with MS and MP extracts used at 400 μg/mL; no apoptotic cells were found with MR extracts. With 7KC used as the positive control, 65% and 98% of apoptotic cells were detected at 24 and 48 h, respectively (Figure 4).

### Evaluation of the effects of *Mentha* extracts on caspase-3 activation and LC3-I to LC3-II conversion on RAW 264.7 cells

**Figure 5.**
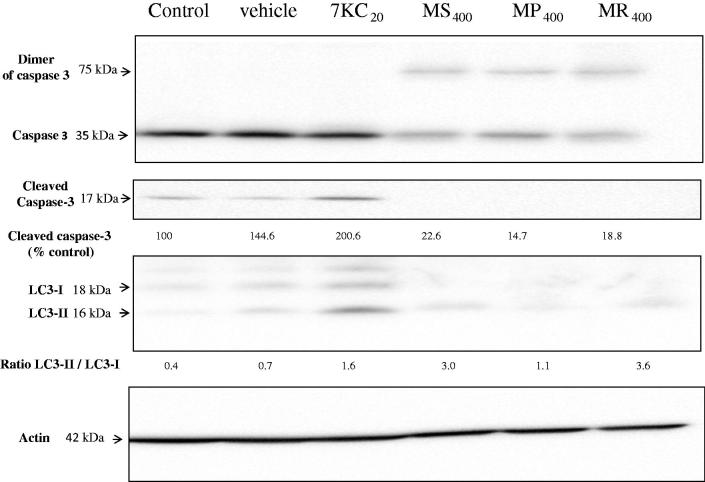
Effects of *Mentha* extracts on caspase-3 activation and LC3-I to LC3-II conversion. The ability of the extracts from three Algerian *Mentha* species (*M. spicata* L. (MS)*, M. pulegium* L. (MP), and *M. rotundifolia* (L.) Huds (MR): 400 μg/mL final concentration, 24 h) to trigger caspase-3 activation and LC3-I to LC3-II conversion were evaluated by western blotting on RAW264.7 cells. 7-Ketocholesterol (7KC; 20 μg/mL) was used as the positive control; vehicle control for 7KC corresponds to ethanol (0.1%). Data shown are representative of three independent experiments. Actin was used as the internal standard. The numbers given above the blots represent the values of the level of uncleaved and cleaved caspase-3, and the LC3-II/LC3-I ratio. The experiment was done twice and gave similar results.

To determine the precise effect of *Mentha* extracts (400 μg/mL, 24 h) on apoptosis and autophagy, the presence of cleaved caspase-3, and the conversion of microtubule-associated protein light chain 3 (LC3-I) to LC3-II (evaluated by the ratio LC3-II/LC3-I) were studied by Western blotting. With the different mint extracts used, no cleaved caspase-3 was observed. However, with the different mint extracts considered, a dimeric form of caspase-3 was found: a band of approximately 75 kDa was revealed (Figure 5).

With 7KC, increased levels of cleaved caspase-3 and an increased LC3-II/LC3-I ratio, which are specific criteria of apoptosis and autophagy, respectively, were observed, thus underlining the ability of 7KC to simultaneously activate apoptosis and autophagy (Figure 5).

## Discussion

Increasing attention is being paid to the study of natural products, used in traditional medicine, which may counteract the detrimental effects of environmental toxic compounds and/or prevent multiple human diseases. In this context, different medicinal plants have been reevaluated and recognized as valuable sources of nutraceuticals (Kilani-Jaziri et al. 2014). The present study provides evidence that some *Mentha* species extracts (*M. spicata; M. pulegium* and *M. rotundifolia*) are active on the apoptotic and autophagic pathways on murine and human monocytic cells. So far, the biological activities of *Mentha* extracts have been reported especially on tumor cells. They concern cell growth, mitochondrial effects, and the induction of apoptosis.

To date, several studies have shown the effect of *Mentha* extracts on cell growth. Most of these have shown the ability of such extracts to reduce the number of several types of tumour cells. Hussain et al. (2010) showed the effect of increasing amounts of *Mentha* essential oils on the cell proliferation of two human cancer cell lines (MCF-7 and LNCaP). The inhibitory effect of *Mentha* essential oils on cell viability ranged from 91 to 97% at 0.5 mg/mL. The cytotoxic effects of *M. spicata* aqueous extract on cancer cell lines have been revealed *in vitro* (Hajighasemi et al. 2011). In agreement with these previous data, *M. spicata* and *M. rotundifolia* extracts studied here at high concentrations could be cytotoxic for tumour cells since they decrease cell growth and viability at concentrations near to IC_50_ values. This could be due to their chemical compositions. Carotenoids such as β-carotene and β-cryptoxanthin, as well as flavonoids such as apigenin and luteolin in mint have been found to have anticancer properties, and could be involved in cell growth inhibition and/or mitochondrial dysfunctions. Bandyopadhyay et al. (2008) reported that mint leaves have powerful anti-angiogenesis effects and can kill tumour cells. In one of our previous studies, we reported the predominance of flavonoids in MS and MR extracts (Brahmi et al. 2015), which could also be responsible for their effect on cell growth. Some studies have shown that these compounds are able to influence a variety of cell functions by modulating cell signalling, altering proliferation and inducing cytotoxicity in cancer cell lines. Moreover, flavonoids showed cytotoxic effects on various human cell lines, such as leukaemia cells and ovarian cancer cells (Kilani-Jaziri et al. 2014). In the present study, all of the extracts inhibited cell growth (as shown by phase-contrast microscopy, crystal violet and MTT tests), whereas only MS and MR extracts showed cytotoxic effects in all of the tests. This finding supports the notion that this activity depends on the species. The low cytotoxicity exhibited by the *M. pulegium* extracts supports that the compounds present in this plant are weakly cytotoxic. So, the characteristics and the chemical profiles of the compounds present in the species could influence their cytotoxicity.

As *Mentha* extracts at high concentrations inhibit cell proliferation, we determined whether this effect could be related to mitochondrial dysfunction, especially the loss of transmembrane mitochondrial potential (ΔΨm) monitored by flow cytometry using DiOC_6_(3). This analysis showed that high concentrations of the extracts of the three *Mentha* species caused a loss of transmembrane mitochondrial potential with the maximum percentage of cells with depolarized mitochondria reaching 75% with the *M. spicata* extract. It can therefore be suggested, that mitochondrial dysfunctions can be involved as either a primary or secondary event in mint extract-induced cell-growth inhibition. Indeed, *Mentha* extracts contain a mixture of compounds which can promote the loss of ΔΨm. However, this does not exclude the possibility that they may also contain compounds that prevent the loss of ΔΨm. Indeed, our previous work demonstrated that rosmarinic acid was the major phenolic acid present in the three *Mentha* species considered (MS, MP, and MR) (Brahmi et al. 2014, Brahmi et al. 2015). Kim et al. (2005) studied the inhibitory effects of rosmarinic acid on adriamycin-induced apoptosis in H9c2 cardiac muscle cells. They found that adriamycin reduced mitochondrial membrane potential, and that pretreatment with rosmarinic acid prevented this decrease. Targeting mitochondria with *Mentha* extracts able to prevent loss of ΔΨm can be of interest for anti-aging purposes, with the main applications focused on neurodegenerative diseases and cardioprotection, while molecules triggering the loss of ΔΨm may have several interests in preventing acute or chronic inflammatory diseases or cancer as ΔΨm loss is closely connected to different forms of cell death such as apoptosis (Forbes-Hernandez et al. 2014).

Surprisingly, following staining with PI, which enters dead cells and cells with damaged membranes, a rapid accumulation of the dye was observed in all cells with all extracts, even though not all mint extracts were strongly cytotoxic according to MTT, crystal violet, and DiOC_6_(3) tests. As these extracts are rich in apolar compounds (essential oils), it is suggested that their interaction with cytoplasmic membrane lipids may favour PI accumulation in the cells independently of cell death but as a consequence of cytoplasmic membrane damage. Of note, in agreement with our observation, Cox et al. (2000) reported that exposing micro-organisms to tea tree oil increased the permeability of bacterial cytoplasmic and yeast plasma membranes as indicated by the uptake of PI, to which the cell membrane is normally impermeable.

Since mint extracts induce mitochondrial dysfunction revealed with the MTT test and by staining with DiOC_6_(3), the concentrations of the extracts which caused a loss of ΔΨm were tested in order to evaluate caspase-3 activation associated with apoptosis induction and the conversion of microtubule-associated protein light chain 3 (LC3-I) to LC3-II, which is a criterion of autophagy.

Caspase cascade activation is critical for apoptotic initiation in many biological systems (Kim et al. 2005). To determine whether caspase-3 activation is a factor in the cytotoxicity of mint extracts, we monitored their catalytic activity by Western blotting. According to our results, mint extracts do not induce the cleavage of caspase-3. On the contrary, we noted the formation of a dimer of caspase-3 of about 75 kDa, suggesting the presence of kosmotrope protein structures in mint extracts (Pop et al. 2006; Hughes et al. 2009). This kosmotrope protein structure could stabilize the uncleaved and inactive caspase-3 in its dimeric form. However, as cells with condensed and/or fragmented nuclei, typical of apoptosis (Lizard et al. 1995), were observed under treatment with mint extracts, our data support the notion that apoptotic pathway(s) independent of caspase-3 may be activated. It is noteworthy, that only MS and MR extracts were also able to promote the conversion of LC3-I to LC3-II, suggesting the activation of autophagy. Our data therefore support the notion that some mint extracts are able to interact with the apoptotic and the autophagic pathway. These properties constitute an additional argument supporting the hypothesis that mint extracts may have some benefits in major diseases, where there is a need to trigger cell death. According to Forbes-Hernandez et al. (2014), dietary compounds, mainly those of vegetable origin, have been shown to target signalling intermediates in apoptosis-inducing pathways. In cancer cells, dietary compounds could protect against disease progression by enhancing the elimination of initiated precancerous cells and therefore acting like pro-apoptotic agents. On the contrary, in healthy cells, these compounds usually protect against apoptosis, thus acting as anti-apoptotic elements.

In mint extracts, the pro-apoptotic properties might be possibly due to the presence of caffeic and rosmarinic acids, along with other compounds. The pro-apoptotic effect of ‘caffeic acid phenetyl ester (CAPE)’ has been demonstrated in damaged cells (Forbes-Hernandez et al. 2014). In contrast, these compounds may inhibit apoptosis in normal cells. Rosmarinic acid-induced inhibition of apoptosis is associated with the regulation of Mn-superoxide dismutase and glutathione, and on their scavenging of free radicals in cardiac muscle cells (Kim et al. 2005).

## Conclusion

Altogether, our data bring new information on the effects of aqueous ethanolic mint extracts on cell growth and cell death in monocytic cells, and they establish that, depending on the mint extract considered, it is possible to trigger apoptosis and autophagy. Therefore, mint extracts have strong biological activity. In the present study, all of the mint extracts studied (MS, MP, and MR) were able to reduce cell growth, at relatively high concentrations (200–400 μg/mL), and to simultaneously trigger a loss of ΔΨm and to alter cytoplasmic membrane integrity. However, only MS and MR were able to trigger apoptosis and autophagy. Our data therefore support the notion that some mint extracts can simultaneously have pro-apoptotic and pro-autophagic properties which could be of interest to prevent diseases where there is a need to activate these processes. Although additional investigations are required to establish the molecular mechanisms of each of the mint extracts studied, our data show that mint extracts interact with the apoptotic and autophagic pathways. Complementary *in vivo* studies, conducted in appropriate animal models, are now required to determine the most appropriated therapeutic strategies allowing various mint extracts to be used efficiently in major human diseases.
